# Single-Center Experience With Epigenetic Treatment for Juvenile Myelomonocytic Leukemia

**DOI:** 10.3389/fonc.2020.00484

**Published:** 2020-04-09

**Authors:** Andra Marcu, Andrei Colita, Letitia Elena Radu, Cristina Georgiana Jercan, Ana Maria Bica, Minodora Asan, Daniel Coriu, Alina Daniela Tanase, Carmen C. Diaconu, Cristina Mambet, Anca Botezatu, Sergiu Pasca, Patric Teodorescu, Gabriela Anton, Petruta Gurban, Anca Colita

**Affiliations:** ^1^Department of Stem Cell Transplantation, Fundeni Clinical Institute, Bucharest, Romania; ^2^Department of Pediatrics, Carol Davila University of Medicine and Pharmacy, Bucharest, Romania; ^3^Department of Stem Cell Transplantation, Coltea Hospital, Bucharest, Romania; ^4^Department of Hematology, Carol Davila University of Medicine and Pharmacy, Bucharest, Romania; ^5^Department of Hematology, Titu Maiorescu University of Medicine, Bucharest, Romania; ^6^Cellular and Molecular Pathology Department, Stefan S Nicolau Institute of Virology, Bucharest, Romania; ^7^Molecular Virology Department, Stefan S Nicolau Institute of Virology, Bucharest, Romania; ^8^Research Center for Functional Genomics and Translational Medicine, Iuliu Hatieganu University of Medicine and Pharmacy, Cluj Napoca, Romania; ^9^Department of Hematology, Iuliu Hatieganu University of Medicine and Pharmacy, Cluj Napoca, Romania; ^10^Department of Hematology, Ion Chiricuta Clinical Cancer Center, Cluj Napoca, Romania; ^11^Personal Genetics-Medical Genetics Center, Bucharest, Romania

**Keywords:** juvenile myelomonocytic leukemia, mutation, epigenetics, methylation, azacytidine, hematopoietic stem cell transplantation

## Abstract

**Background:** Juvenile myelomonocytic leukemia (JMML) is a rare myelodysplastic/myeloproliferative neoplasm diagnosed in young children, characterized by somatic or germline mutations that lead to hyperactive RAS signaling. The only curative option is hematopoietic stem cell transplantation (HSCT). Recent data showing that aberrant DNA methylation plays a significant role in pathogenesis and correlates with clinical risk suggest a possible benefit of hypomethylating agents (HMA) in JMML treatment.

**Aim:** The aim is to report the results of HMA-based therapy with 5-azacytidine (AZA) in three JMML patients treated in a single center, non-participating in EWOG-MDS study.

**Methods:** The diagnosis and treatment response were evaluated according to international consensus criteria. AZA 75 mg/m^2^ intravenous (i.v.) was administered once daily on days 1–7 of each 28-day cycle. All patients were monitored for hematologic response, spleen size, and evolution of extramedullary disease. Targeted next generation sequencing (NGS) were performed after the 3rd AZA cycle and before SCT to evaluate the molecular alterations and genetic response.

**Results:** Three patients diagnosed with JMML were treated with AZA (off-label indication) in Pediatric Department of Fundeni Clinical Institute, Bucharest, Romania between 2017 and 2019. There were two females and one male with median age 11 months, range 2–16 months. The cytogenetic analysis showed normal karyotype in all patients. Molecular analysis confirmed KRAS G13D mutation in two patients and NRAS G12D mutation in one patient. The clinical evaluation showed important splenomegaly and hepatomegaly in all 3 pts. One patient received AZA for early relapse after haploidentical HSCT and the other two patients received upfront AZA, as bridging therapy before HSCT. After HMA therapy, 2/3 patients achieved clinical partial response (cPR), 1/3 had clinical stable disease (cSD) and all had genetic stable disease (gSD) after 3 cycles and were able to receive the planned HSTC. One patient achieved clinical and genetic complete response before HSCT. During 22 cycles of AZA there were only four adverse events but only one determined dose reduction and treatment delay.

**Conclusion:** Our data show that AZA monotherapy is safe and effective in controlling disease both in upfront and relapsed patients in order to proceed to HSCT.

## Introduction

Juvenile myelomonocytic leukemia (JMML) is a rare myeloproliferative/myelodysplastic neoplasm of early infancy and childhood defined by an excessive production of mature and immature myeloid cells, predominantly of monocytic and granulocytic lineages (1, 2). Therapeutic approaches range from watchful monitoring to allogeneic hematopoietic stem cell transplantation (HSCT) performed in early stages (3). Clinical symptoms result from hematopoietic insufficiency and leukemic infiltration of various organs, such as spleen, liver, skin, lung, and gastrointestinal tract (4). Conventional cytogenetics studies indicate monosomy 7 in up to 25% of JMML patients and other abnormalities in 10% of cases. However, a normal karyotype is diagnosed in about two-thirds of patients (5–7). Strikingly, many JMML children with a normal karyotype exhibit an elevated level of fetal hemoglobin (HbF) (8).

The genetic landscape of JMML is dominated by somatic or germline mutations that lead to hyperactive RAS signaling (9). Five molecular alterations of RAS pathway were described in association with five JMML genetic subtypes, that have distinct clinical and hematological features (1). Three subtypes, covering 55–60% of patients, involve heterozygous somatic activating mutations in *PTPN11, NRAS*, and *KRAS* genes (1, 9). The other two subtypes (20–30% of JMML cases) occur in patients with underlying constitutional diseases caused by germline mutations in the RAS pathways (RASopathies): neurofibromatosis type 1 (NF1) and Noonan-like “CBL syndrome,” respectively. Hematological disorders develop due to the acquired loss of heterozygosity of the constitutionally affected *NF1* or *CBL* tumor suppressor genes in the hematopoietic progenitors (4, 10, 11). In rare cases of JMML that lack the above-mentioned mutations, heterozygous somatic *RRAS* mutations have been reported as disease drivers (12). In addition to RAS pathway mutations, whole-exome sequencing has identified secondary molecular events in ~50% of patients. These alterations, including also mutations in epigenetic regulation genes (*EZH2, ASXL1, DNMT3A*), might impact clinical outcome and therapeutic decisions (1). Interestingly, JMML displays a unique linear pattern of disease progression. As shown previously, all mutations present at diagnosis are acquired by a single dominant clone that is consistently detected at relapse. Also, Stieglietz et al. have reported that the number of somatic mutations identified at diagnosis influences the survival rate, while the type of RAS pathway mutations that does not represent an independent prognostic factor (13).

Aberrant DNA methylation is another factor related to adverse clinical outcome in JMML patients. European Working Group on MDS in Childhood (EWOG-MDS) published in 2011 a study on 127 JMML patients, evaluating DNA methylation at 14 loci through quantitative mass spectrometry, describing CpG island hypermethylation in the *BMP4, CALCA, CDKN2B*, or *RARB* promoter regions as being the best predictor of relapse after HSCT (14). Similarly, different other studies showed that DNA hypermethylation is connected to clinical risk (15–18). Recently, three JMML epigenetic subgroups based on DNA methylation profiling were identified. The low methylation cluster is defined by the presence of Noonan syndrome, *CBL* mutations and the majority of *NRAS* mutations, having a favorable outcome. The intermediate methylation cluster consists of patients with numeric aberration of chromosome seven (monosomy) and somatic *KRAS* mutations. The high methylation subgroup includes patients displaying somatic *PTPN11* mutation, low platelet count, elevated HbF, and diagnosis established after the age of two. This subgroup was specifically associated with a higher rate of disease relapse and dismal prognosis (2).

The aberrant DNA methylation patterns described in JMML create a premise for the clinical use of hypomethylating agents, such as 5-azacytosine (azacytidine) and 2′-deoxy-5-azacytidine (decitabine). Once entering the cell, azacytidine is activated through consecutive ATP-dependent phosphorylation steps. Eighty to ninety percentage of azacytidine is incorporated into RNA leading to apoptosis (19) 10–20% of azacytidine is integrated into DNA, permanently inhibiting DNA methyltransferase, suppressing its function, and causing its degradation. Methylation marks are lost during DNA replication, reversing silenced tumor suppressor genes and recovering proliferation, and apoptosis control (20–22). Azacytidine induces specific immune responses through azacytidine-induced immune genes and inhibition of regulatory T cells (23–25).

## Case Presentations

Two girls and one boy, median age 11 months, range 2–16 months, were diagnosed and treated with azacytidine (off label indication) in the Pediatric Department of Fundeni Clinical Institute, Bucharest, Romania, between 2017 and 2019. On admission, mean hemoglobin (Hb) was 8,8 g/dl (range 7,1–10,2 g/dl), mean white blood cell (WBC) count was 22,576 × 10^9^/L (range 19,75–28,21 × 10^9^/L), mean monocyte count was 7,1 × 10^9^/L (range 6,2–8,4 × 10^9^/L) and mean platelet count was 65 × 10^9^/L (range 46–77 × 10^9^/L). All patients presented with blasts in the bone marrow 4–15% (mean 10%), while only 1 patient presented with 5% myeloid blasts in peripheral blood. The cytogenetic analysis showed normal karyotype, while targeted next-generation sequencing (NGS) revealed *KRAS* G13D mutation in two patients and *NRAS* G12D mutation in one patient. All patients had important splenomegaly, with mean spleen size of 7 cm (range 3–10 cm) under the costal margin and hepatomegaly with mean liver size of 5 cm (range 4–8 cm) under costal margin. One patient received azacytidine for early relapse after hematopoietic stem cell transplantation (HSCT) and the other two received upfront azacytidine, as bridging therapy before HSCT (Table 1).

**Table 1:** Response to azacytidine in children with JMML.

	**Disease status**	**Sex** **Age** **(months)**	**Cytogenetics**	**Mutational group**	**No. of AZA cycles**	**Concomitant** **treatment**	**Response after 3rd AZA**		**Response before HSCT**		**HSCT** **number type**	**Status** **Post-transplant**	**Follow-up (months)**
							**Clinic**	**Genetic**	**Clinic**	**Genetic**			
Case 1	Relapse after 1st HSCT	M 11	46, XY FISH−7/del7q negative	NRAS	8	6MP	cPR	gSD	cPR	gSD	2 Haplo MUD	Alive gCR	46
Case 2	*De novo* JMML	F 2	46, XX FISH−7/del7q negative	KRAS	8	no	cPR	gSD	cCR	gCR	1 MUD	Alive gCR	14
Case 3	*De novo* JMML	F 16	46, XX FISH−7/del7q negative	KRAS	6	no	cSD	gSD	cPD	gSD	1 MSD	Alive gSD	12

*6MP, 6 mercaptopurin; cPR, clinical partial remission; cSD, clinical stable disease; cCR, clinical complete remission; cPD, clinical progressive disease; gCR, genetic complete response; gSD, genetic stable disease; MUD, matched unrelated donor; MSD, matched sibling donor; mo, month; F, female; M, male; haplo, haploidentical HSCT*.

JMML diagnosis and therapy response were evaluated according to international consensus criteria (Table 2) (26). Azacytidine 75 mg/m^2^ i.v. was administered once daily, on days 1–7 of each 28-day cycle. All patients were monitored for hematologic response, spleen size, and evolution of extramedullary disease. NGS at diagnosis and real-time PCR were performed in order to identify the molecular alterations and assess the genetic response after first three cycles and pre-HSCT. Patients were monitored for adverse events (AEs).

**Table 2:** Variables for evaluation of response to therapy in JMML (26).

**Variables for response**		**Definition of response**		**Definition of disease** **progression or relapse** **(applicable to all patients)**
	**Assessment of CR and PR is feasible if the following are present before therapy**	**CR**	**PR**	**PD**
1) WBC count	>20 × 10^9^/L	3.0–15.0 × 10^9^/L	Decreased by ≥50% over pre-treatment but still over >15 × 10^9^/L	Increase by ≥50% and ≥20 × 10^9^/L
2) Myeloid and erythroid precursors and blasts in PB^*^	≥5%	0–1%	Decreased by ≥50% over pre-treatment but still ≥ 2%	Increase from the baseline: <5%: ≥50% increase and ≥5%≥5%: ≥50% increase of total % of myeloid and erythroid precursors and blasts
3) Platelet count	<100 × 10^9^/L	≥100 × 10^9^/L	For patients starting with ≥ 20 × 10^9^/L platelets: absolute increase of ≥30 × 10^9^/L For patients starting with <20 × 10^9^/L platelets: increase by ≥100% and >20 × 10^9^/L	Development of transfusion dependency or, if patients have the baseline of the platelet count ≥30 × 10^9^/L, decrease by ≥100% and <100 × 10^9^/L
4) BM blasts	≥5%	<5%	Decreased by ≥50% over pre-treatment but still ≥5% baseline	Increase from baseline; <5%: ≥50% increase and ≥5%≥5%: ≥50% increaseof BM blasts
5) Spleen size Clinical evaluation	≥2 cm under	No splenomegaly	50% decrease by cm under the costal margin	Increase by ≥100% if baseline <4cm from under the costal margin≥50% if baseline 5-10 cm>30% if baseline >10 cm
Ultrasonography	Length of spleen≥150% of upper limit of normal range	No splenomegaly	>25% decrease by length, but still splenomegaly	Increase by≥25% of length
6) Extramedullary disease^#^	Extramedullary leukemic infiltration	No evidence of extramedullary leukemic infiltration in any organ		Worsening or new lesions of extramedullary leukemic infiltration
7) Cytogenetic response	Somatic cytogenetic abnormality detected	Normal karyotype		Reappearance or additional acquirement of cytogeneticabnormalities
8) Molecular response	Somatic genetic anomalies detected^**^	Absence of somatic genetic anomalies		Reappearance or additional acquirement of JMML-specificsomatic gene abnormalities
9) Chimerism response (only for patients after HSCT)	>15% autologous cells after allo-HSCT	Complete donor chimerism		50% increase and >5% increase of autologous cells and >5%

*CR, complete response; PR, partial response; PD, progressive disease; WBC, white blood cell; PB, peripheral blood; BM, bone marrow*.

* *Myeloid precursors include promyelocytes, myelocytes and metamyelocytes. The myeloid and erythroid precursors and blasts in PB are given as percentage of the total nucleated cells in PB (WBC including erythroblasts)*.

** *In NF-1, PTPN11, NRAS, KRAS, or CBL, thus the mutations are thought to be initiating. In patients with germ-line NF-1, PTPN11 or CBL mutation, only acquired mutations can be evaluated for response and relapse after therapy. The germ-line mutation remains even if patients achieved complete molecular response*.

# *Extramedullary disease includes infiltration of skin, lung, and, very rarely, cranial nerves or central nervous system*.

The first case is of an 11-month-old boy diagnosed with *NRAS*-JMML, treated with 6-mercaptopurine and low-dose cytarabine, while searching for an HLA compatible donor for an allogeneic HSCT. Since the donor was not available at clearance timepoint and considering the emergency for transplantation, he underwent a haploidentical HSCT (haplo HSCT) from his father, with melphalan-fludarabine conditioning regimen, post-transplant cyclophosphamide, and immunosuppressive therapy for graft-vs. -host disease (GvHD) prophylaxis. After engraftment, the chimerism analysis performed on day +19 showed mixed results (53% chimerism from donor). Despite stopping immunosuppression and infusion of donor lymphocytes (DLI), followed by skin and gastrointestinal grade 4 GvHD and severe lung disease, there was a progressive loss of donor cells. Therapy with azacytidine was initiated while a new work-up for second HSCT was started. The infant achieved clinical partial remission (cPR) after three courses of azacytidine and maintained the same status after eight courses. After 1 year, a 9/10 unrelated donor HSCT was performed, with busulfan-cyclophosphamide-melphalan-ATG conditioning. Chimerism analysis on day +24 showed 100% donor cells. He is now at 2 years after 2nd transplant, with very good clinical condition, no chronic GvHD, normal blood count, full donor chimerism, and full immune recovery.

The second case is of a 2-month-old girl diagnosed with *KRAS*-JMML, who was started in first line therapy with azacytidine. She tolerated azacytidine courses very well, without hematological toxicities. After three courses, she obtained cPR (normal WBC, absent myeloid/erythroid precursors or blasts in PB, normal PLT count, no blasts in BM, more than 50% reduction in spleen and liver size) and complete clinical remission (cCR) after eight courses. Targeted next-generation sequencing (NGS) revealed the presence of *KRAS* G13D mutation with 21% variant allele frequency (VAF) at diagnosis. Molecular monitoring by real-time PCR indicated a decrease of mutational load after three courses and genetic complete remission (gCR) after eight courses. 10/10 MUD HSCT, with thiotepa-treosulfan-fludarabine-ATG conditioning has been performed.

The third case is of a 16-month-old girl with *KRAS*-JMML, who was started on upfront azacytidine. The patient's first clinical and hematological abnormalities were noted at 6 months, but the diagnosis was made 10 months later, when she presented with massive hepato-splenomegaly and respiratory manifestations due to leukemic infiltration. Targeted NGS at diagnosis identified *KRAS* G13D mutation with a VAF of 38%. She received six courses of azacytidine as bridging therapy for matched sibling donor HSCT. Evaluation after first three courses showed clinical stable disease (cSD) (normal WBC, no blasts in PB or in the BM, but the patient still presented massive hepato-splenomegaly, and thrombocytopenia). She developed pneumonia complicated with lung abscess and received antibiotic treatment for 28 days, with 2 weeks delay in azacytidine administration, and loss of therapeutic response. After six courses of azacytidine we noted clinical progressive disease (cPD) based on the development of platelet transfusion dependency and increase of spleen size after initial reduction. The molecular monitoring confirmed gSD. Matched sibling donor HSCT with thiotepa-treosulfan-fludarabine conditioning has been performed.

**Figure 1:**
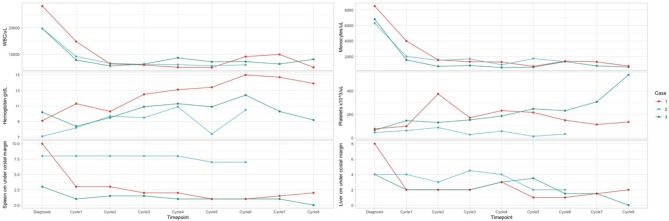
Hematology parameters in dynamics for the patients. Case 2: At diagnosis: KRAS G13D mutation with 21% variant allele frequency (VAF). Molecular monitoring by NGS after 3 courses of therapy indicated a decrease of mutational load to 12%. Molecular monitoring by NGS after 8 courses of therapy indicated complete molecular remission. After HSCT: complete molecular remission. Case 3: At diagnosis: KRAS G13D mutation with a VAF of 38%. Molecular monitoring by NGS after 3 courses of therapy: mutational load 37.5%. Molecular monitoring by NGS at the end of therapy: VAF 35.2%. After HSCT: VAF 2.5%.

We report 4 AEs (fever CTCAE grade 4 – 1 patient, diarrhea CTCAE grade 2 - 2 patients, urticaria and rash grade 2 CTCAE - 1 patient) during 22 cycles of azacytidine, with dose reduction for one course and delay for the next course because of pneumonia. The hematologic monitoring during azacytidine cycles showed normal Hb value, normal WBC, and differential count for all patients, as well as normal PLT count for two of them after the first three courses. No hematologic toxicities were reported in our series (Figure 1). All AEs were managed with standard supportive care and without modifications or delay in azacytidine treatment.

## Discussions

Treatment with HMA aims for clinical and hematological response, transfusion independency, and prolonged survival after HSCT, azacytidine being one of the most used agents. Acknowledging its tolerable toxicity and cytoreductive activity, azacytidine becomes a suitable choice for bridging treatment before HSCT, as well as strategy for second HSCT or palliation (27). JMML is challenging and difficult to treat, the only curative option being HSCT. In its absence, the median survival time from diagnosis is <1year (28).

The published data on HMA therapy in relapsed patients after transplantation is limited to three cases. Cseh et al. (27) reported that azacytidine is correlated with partial response during three cycles in one patient, but all three patients eventually progressed and died. Still, we report a *NRAS*-JMML patient who relapsed after haploidentical HSCT and didn't respond to DLI, despite severe, grade 4 GvHD. He received eight cycles of azacytidine with clinical partial response. After 2nd unrelated HSCT, the patient obtained complete remission, with full donor chimerism. He is alive, fully recovered at 2 years after the 2nd transplant and, in our knowledge, is the first patient showing a favorable response to azacytidine in relapse after HSCT.

Furlan et al. (29) reported a JMML patient with monosomy 7 and *KRAS* mutation who received upfront azacytidine. Good clinical response was documented, with regression of splenomegaly, and monocyte count after the first course of treatment and disappearance of molecular alterations after cycle five (for monosomy 7) and seven (for *KRAS* mutation). The eight courses of azacytidine were followed by allogeneic HSCT with complete remission and disease-free survival at 5 years follow-up. Consequently, further trials were open to evaluate remission response at three cycles of therapy and to establish the remission persistence until transplantation. Cseh et al. (27) mentioned three complete clinical, cytogenetic and/or molecular remissions out of nine patients with JMML who received azacytidine before HSCT. Two of the patients had somatic *PTPN11* mutation and one had *KRAS* mutation, thus showing that certain patients respond to this treatment. Although azacytidine may induce complete clinical, cytogenetic and/or molecular remission before allogeneic HSCT, complete remission has not been maintained without transplant.

Interim analysis of the prospective AZA-JMML-001 study evaluating upfront azacytidine in JMML (30) reported 18 patients with JMML (13 *PTPN11-*, 3 *NRAS-*, 1 *KRAS-*, 1 *NF1-*mutated), classified in DNA methylation classes (MC): high - 11, intermediate (int) – 5 or low for two patients. 11 patients (61%) were in cPR after three cycles of azacytidine, while seven had PD at same treatment stage or prior. All seven patients from the int/low MC and 4/11 from high MC achieved cPR. Seventeen patients received HSCT at a median of 58 days (37–518 days) from last azacytidine dose. Fourteen patients were leukemia-free at a median follow-up of 15.7 months (0.1–31.7 months) after HSCT. Two patients from the high MC relapsed after allograft. 16/18 patients were alive at a median follow-up of 19.8 months (2.6–37.3 months) from diagnosis. One patient discontinued HSCT prior to cycle 3 azacytidine and died from PD. One non-responder patient died from transplant-related causes.

In our case-series we rport cPR at cycle 3 azacytidine in all patients, with gSD. After eight and, respectively, six courses of HMA treatment, we report cCR for one patient, cPR for one patient and cPD for one patient, while genetic response was complete for only one patient before transplantation. Four AEs were reported during 22 cycles of azacytidine, but only one determined dose reduction and treatment delay. Regarding the failure of engraftment for the first transplant for the first patient, it should be mentioned that it was not a failure to engraft, but engraftment with mixed progressive chimerism, followed by complete receptor hematopoiesis, probably in the context of important splenomegaly.

## Conclusions

The heterogeneity of disease evolution in different patients cannot be well-explained yet, though the importance of methylation groups and secondary mutations has already been established.

In accordance with international data, our patient series shows that azacytidine monotherapy is well-tolerated in patients with *de novo* JMML, as well as in patients with relapse after previous treatments, even transplantation. Although the long-term advantage of azacytidine before transplant remains to be fully assessed, responses show it is effective in JMML and provides clinical benefit without severe adverse events.

## Ethics Statement

The study was reviewed and approved by the Ethics Committee of the Fundeni Clinical Institute. Written informed consent was obtained from the minor(s)' legal guardian/next of kin for the publication of any potentially identifiable images or data included in this article.

## Author Contributions

All authors have read and approved the manuscript and contributed to data gathering. AM, AndC, and AncC wrote the manuscript.

### Conflict of Interest

The authors declare that the research was conducted in the absence of any commercial or financial relationships that could be construed as a potential conflict of interest. The reviewer A-AZ declared a shared affiliation, with no collaboration, with several of the authors, PT and SP, to the handling editor at the time of review.
